# The paracetamol metabolite N-acetylp-benzoquinone imine reduces excitability in first- and second-order neurons of the pain pathway through actions on K_V_7 channels

**DOI:** 10.1097/j.pain.0000000000001474

**Published:** 2019-02-08

**Authors:** Sutirtha Ray, Isabella Salzer, Mira T. Kronschläger, Stefan Boehm

**Affiliations:** a Division of Neurophysiology and Neuropharmacology, Center for Physiology and Pharmacology, Medical University of Vienna, Vienna, Austria; b Department of Neurophysiology, Center for Brain Research, Medical University of Vienna, Vienna, Austria

**Keywords:** NAPQI, Membrane excitability, K_V_7 channels, Dorsal root ganglion neurons, Spinal dorsal horn neurons

## Abstract

Paracetamol (acetaminophen, APAP) is one of the most frequently used analgesic agents worldwide. It is generally preferred over nonsteroidal anti-inflammatory drugs because it does not cause typical adverse effects resulting from the inhibition of cyclooxygenases, such as gastric ulcers. Nevertheless, inhibitory impact on these enzymes is claimed to contribute to paracetamols mechanisms of action which, therefore, remained controversial. Recently, the APAP metabolites N-arachidonoylaminophenol (AM404) and N-acetyl-p-benzoquinone imine (NAPQI) have been detected in the central nervous system after systemic APAP administration and were reported to mediate paracetamol effects. In contrast to nonsteroidal anti-inflammatory drugs that rather support seizure activity, paracetamol provides anticonvulsant actions, and this dampening of neuronal activity may also form the basis for analgesic effects. Here, we reveal that the APAP metabolite NAPQI, but neither the parent compound nor the metabolite AM404, reduces membrane excitability in rat dorsal root ganglion (DRG) and spinal dorsal horn (SDH) neurons. The observed reduction of spike frequencies is accompanied by hyperpolarization in both sets of neurons. In parallel, NAPQI, but neither APAP nor AM404, increases currents through K_V_7 channels in DRG and SDH neurons, and the impact on neuronal excitability is absent if K_V_7 channels are blocked. Furthermore, NAPQI can revert the inhibitory action of the inflammatory mediator bradykinin on K_V_7 channels but does not affect synaptic transmission between DRG and SDH neurons. These results show that the paracetamol metabolite NAPQI dampens excitability of first- and second-order neurons of the pain pathway through an action on K_V_7 channels.

## Abstract 2

The paracetamol metabolite NAPQI is revealed to reduce action potential firing in dorsal root ganglion and spinal dorsal horn neurons through activation of K_V_7 channels.

## 1. Introduction

Paracetamol (acetaminophen) is often referred to as the drug that is used most frequently around the globe to treat mild to moderate pain and to reduce fever.^5,17^ It is generally favored over nonsteroidal anti-inflammatory drugs (NSAIDs) as it provokes serious adverse events less frequently. Nevertheless, paracetamol overdose may cause acute liver failure, a toxic effect rarely seen with NSAIDs.^5,17^ Analgesic and unfavorable actions of NSAIDs, such as gastric ulcers, kidney dysfunction, and bleeding, involve inhibition of cyclooxygenases (COX). Because these detrimental effects are not associated with paracetamol, the latter is not viewed as prototypic COX inhibitor. Still, paracetamol has been shown to inhibit COX-1 and COX-2 with half maximal effects at 105 and 26 µM, respectively,^21^ and COX inhibition is frequently inferred as an analgesic mechanism of this drug.^5,19^

In light of uncertainties regarding COX inhibition as paracetamol's most relevant mechanism of action, numerous investigations have searched for alternative explanations. In this respect, 2 paracetamol metabolites have gained particular attention. In humans, between 5% and 15% of a paracetamol dose is metabolized through cytochrome P450 enzymes (mainly CYP2E1 and CYP3A4) to N-acetyl-p-benzoquinone imine (NAPQI), which is known to mediate hepatotoxicity after paracetamol over dosage.^34^ Less than 0.01% of paracetamol is converted to N-arachidonoylphenolamine (AM404) through conjugation of the intermediate product p-aminophenol with arachidonic acid by the enzyme fatty acid amide hydrolase (FAAH).^22^ N-acetyl-p-benzoquinone imine activates TRPA1^1^ and TRPV1^13^ channels, whereas AM404 activates TRPV1^32^ and inhibits Ca_V_3.2 channels.^24^ TRPV1 and Ca_V_3.2 channels in the brain and TRPA1 channels in the spinal cord seem to be involved in the antinociceptive actions of these paracetamol metabolites, because both, intracerebroventricular application of AM404^24,32^ and intrathecal administration of NAPQI,^1^ provide analgesia in rodent models of chemical and thermal pain. Because knock out of either TRPV1^32^ or TRPA1^1^ was sufficient to abolish the antinociceptive action of APAP, it remains unclear whether any of these 2 channels might represent a single relevant target for paracetamol and its active metabolites.

Recently, paracetamol has been reported to exert anticonvulsant actions,^48,49^ whereas NSAIDs such as indomethacin or diclofenac provide proconvulsive effects.^49^ Numerous anticonvulsive agents are used to treat chronic pain, in particular various neuropathic forms of peripheral origin.^14^ Hence, one may expect that dampening of neuronal activity by paracetamol as basis for its anticonvulsive action^48,49^ may also contribute to its analgesic activity. Accordingly, this study investigated whether paracetamol itself or one of its active metabolites, NAPQI and AM404, may affect excitability in first- and second-order neurons of the pain pathway. Our electrophysiological experiments in primary cultures of dorsal root ganglion (DRG) and spinal dorsal horn (SDH) neurons as well as transversal spinal cord slices reveal that NAPQI, but not paracetamol or AM404, facilitates the opening of K_V_7 channels and thereby hyperpolarizes the membrane potential and reduces action potential firing but does not affect synaptic transmission between these neurons.

## 2. Materials and methods

### 2.1. Materials

Paracetamol (acetaminophen, APAP), capsaicin, cytosine arabinoside, kynurenic acid, phosphocreatine, putrescine, progesterone, Na_2_-ATP, Na_4_-EGTA, Na-GTP, and bulk chemicals for electrophysiological experiments in primary neuronal cultures were purchased from Sigma-Aldrich (Vienna, Austria). For recordings in slices, NaCl, sucrose, NaHCO_3_, glucose, and KCl were procured from Carl Roth (Karlsruhe, Germany); CaCl_2_, KH_2_PO_4_, and MgSO_4_ were purchased from Merck (Vienna, Austria). N-(4-Hydroxyphenyl)-5Z, 8Z, 11Z, 14Z-eicosatetraenamide, N-(4-hydroxyphenyl) arachidonoylamide (AM404) was purchased from Enzo Biochem (New York), amphotericin B from PanReac AppliChem (Darmstadt, Germany), bradykinin, (2E)-N-(2,3-dihydro-1,4-benzodioxin-6-yl)-3-[4-(1,1-dimethylethyl) phenyl]-2-propenamide (AMG 9810), and 2-(1,3-dimethyl-2,6-dioxo-1,2,3,6-tetrahydro-7H-purin-7-yl)-N-(4-isopropylphenyl) acetamide (HC 030031) from Tocris Bioscience (Bristol, United Kingdom), cyano-2,3-dihydroxy-7-nitroquinoxaline (CNQX) from Biotrend (Cologne, Germany) or Abcam (Cambridge, United Kingdom), linopirdine dihydrochloride from Hello Bio (Princeton), N-acetyl-p-benzoquinone imine (NAPQI) from Szabo-Scandic (Vienna, Austria), and 10,10-bis(4-pyridinylmethyl)-9(10H)-anthracenone (XE991) was purchased from Alomone Labs (Jerusalem, Israel). Insulin–transferrin–sodium selenite supplement was obtained from Roche (Mannheim, Germany). Tetrodotoxin was obtained from Latoxan (Valence, France).

### 2.2. Primary neuronal cultures

Pregnant rats or rat dams plus litter were purchased from the Division of Laboratory Animal Science and Genetics at the Department of Biomedical Research of the Medical University of Vienna and were kept in a Scantainer (Scanbur, Karlslunde, Denmark) under standard conditions (12-hour light–dark cycle, 20-25°C, 40%-70% humidity, food, and water ad libitum) until sacrificed. Our methods of DRGs and spinal dorsal horn (SDH) culture preparation have been described in detail before.^27^ For the former, 10- to 14-day-old Sprague–Dawley rat pups of both sexes were decapitated after CO_2_ asphyxia, and the ganglia along the entire length of the spinal cord were collected. These were then incubated in collagenase (1.5 mg·mL^−1^; Sigma-Aldrich, St. Louis, MO) and dispase (3 mg·mL^−1^; Boehringer Mannheim, Vienna, Austria) for 30 minutes followed by 0.25% trypsin (Worthington, Lakewood, NJ) for 10 minutes at 37°C. After gentle trituration using fire polished pipettes, cells were resuspended in Dulbecco's Modified Eagle Medium containing 4.5-g·L^−1^ glucose, 10-mg·L^−1^ insulin, 25,000-IU·L^−1^ penicillin, 25-mg·L^−1^ streptomycin (Sigma-Aldrich), and 50-μg·L^−1^ nerve growth factor (Biomedica, Vienna, Austria). A total number 50,000 cells were seeded into wells created by glass rings with an inner diameter of 10 mm and placed in the center of 35-mm culture dishes coated with poly D-lysine (Sigma-Aldrich). After 2 hours, the medium was supplemented with 5% heat-inactivated fetal calf serum (FCS; Biochrom, Berlin, Germany), and the glass rings were removed. The medium was exchanged completely the day after preparation. The cultures were kept at 37°C in a humidified 5% CO_2_ atmosphere for up to 3 days, during which recordings were made.

Spinal dorsal horn cultures were prepared from p0 to p1 Sprague–Dawley rat pups of both sexes, which were killed by decapitation after CO_2_ asphyxia. The entire spinal cord was dissected after ventral laminectomy and longitudinally cut into 2 halves. The meninges were removed, and the dorsal horn was dissected from each half, cut into small pieces, and incubated in papain (1 mg·mL^−1^ in L-15 Leibovitz medium supplemented with 1-mM kynurenic acid, Sigma-Aldrich) for 30 minutes at 37°C. The tissue was further dissociated by trituration in DMEM containing 10% heat-inactivated FCS and 5-mg·mL^−1^ insulin, 5-mg·mL^−1^ transferrin, 5-mg·mL^−1^ Na-selenite, 18-mg·mL^−1^ putrescine, 10-nM progesterone, 2-mM MgCl_2_, 25,000-IU·L^−1^ penicillin, and 25-mg·L^−1^ streptomycin. The cells were seeded as described above for DRG cultures. The medium in the dishes was replaced by antibiotic-free medium on the day after preparation, and 1 µM cytosine arabinoside was added on the third day to prevent further proliferation of non-neuronal cells. The cultures were kept at 37°C in a humidified 5% CO_2_ atmosphere for up to 3 weeks, and all recordings were made from day 7 to day 21 after preparation.

### 2.3. Cell lines and transient transfection

Cultured tsA201 cells were propagated in DMEM with 10% FCS. Cells were maintained in a humidified atmosphere of 5% CO_2_ at 37°C and transiently transfected using Turbofect (Thermo Fisher, Rockford, IL) and plasmids coding for human K_V_7.2 and K_V_7.3 plus a plasmid coding for enhanced green fluorescent protein as described previously.^45^ Electrophysiological experiments were performed on fluorescent cells 48 hours after transfection.

### 2.4. Spinal cord slice preparation

All surgical procedures and electrophysiological recordings were performed in a manner similar to that described elsewhere.^20^ In short, male Sprague–Dawley rats (20-25 days old) were deeply anaesthetized using isoflurane and were sacrificed by decapitation. The spinal cord was exposed by laminectomy and transferred into ice-cold preoxygenated incubation solution of following composition (in mM): NaCl (95), KCl (1.8), KH_2_PO_4_ (1.2), CaCl_2_ (0.5), MgSO_4_ (7), NaHCO_3_ (26), glucose (15), and sucrose (50) and was oxygenated with a 95% O_2_ and 5% CO_2_ mixture; pH 7.4, measured osmolarity 310 to 320 mOsmol·L^−1^. The dura mater and the ventral roots were removed. Five hundred- to 600-µm-thick transverse spinal cord slices (L4-L6) each with attached dorsal roots (10-15 mm) were cut on a microslicer (DTK-1000; Dosaka, Kyoto, Japan). The slices were incubated for 30 minutes at 32°C and then stored at room temperature (∼21°C) in oxygenated incubation solution until further use.

### 2.5. Electrophysiological experiments in primary neuronal cultures and cell lines

Electrophysiological recordings were performed at room temperature (20-24°C) using an Axopatch 200B amplifier plus Digidata 1440 and pCLAMP 10.4 hardware and software (Molecular Devices, Sunnyvale, California). All experiments were performed in perforated patch clamp mode. Currents through K_V_7 channels were low-pass filtered at 2 kHz and digitized at 10 kHz. Recordings in current clamp were low-pass filtered at 10 kHz and digitized at 40 kHz. Patch electrodes were pulled from borosilicate glass capillaries (GB150-8P; Science Products, Hofheim, Germany) with a Sutter P-97 puller (Sutter Instruments, Novato, CA) using a trough filament (FT330B, Science Products), which achieved pipette resistances between 1 and 3 MΩ. Pipettes were front-filled with internal solution and then back-filled with the same solution containing 500 μg·mL^−1^ amphotericin B. After establishing a GΩ seal, the series resistance was monitored until it stabilized below 20 MΩ, typically after 20 to 30 minutes. The internal solution for all electrophysiological recordings in cultured neurons and transfected cells contained the following (in mM): K_2_SO_4_ (75), KCl (55), MgCl_2_ (8), and HEPES (10), adjusted to pH 7.4 with 1 M KOH. The external solution for all electrophysiological recordings in cultured neurons and transfected cells contained (in mM) the following: NaCl (140), glucose (20), HEPES (10), CaCl_2_ (2.5), MgCl_2_ (2), KOH (3), and NaOH (2), which results in pH 7.4. Tetrodotoxin (0.5 μM) was added to the bath solution for all voltage-clamp recordings in neurons. In addition, the following synaptic blockers were added to the bath solution for all recordings in SDH cultures: kynurenic acid (1 mM) and picrotoxin (30 μM). The solutions resulted in a liquid junction potential of 8 mV, which was corrected for. Stock solutions of APAP, AM404, NAPQI, and picrotoxin were prepared in DMSO, those of XE991, and linopirdine in deionized water. Stock solution of capsaicin was prepared in ethanol and diluted further in external solution; stock solution of kynurenic acid was prepared in 0.1 M NaOH. The appropriate amount of DMSO (solvent) was supplemented in control solutions. Drugs were applied with an OctaFlow perfusion system (ALA Scientific Instruments Inc, Westbury, NY), which allows for a complete exchange of solutions surrounding the patched cell within 100 ms.

**Figure 1.**
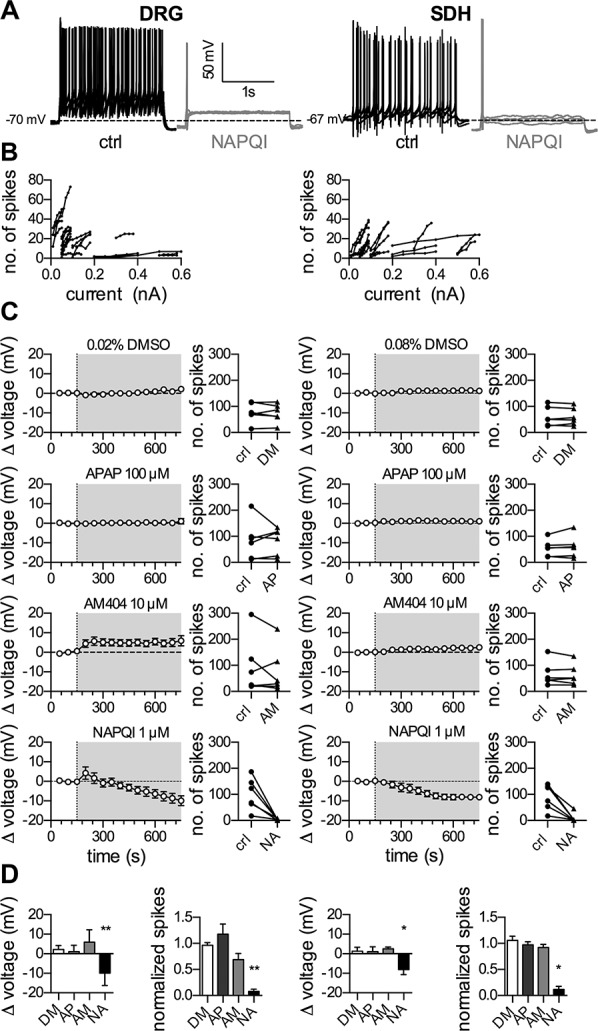
Effects of APAP, AM404 and NAPQI on membrane voltage and excitability of rat DRG and SDH neurons. Dissociated rat dorsal root ganglion (DRG) and spinal dorsal horn (SDH) neurons were recorded in perforated current-clamp mode. Baseline values for membrane voltage were recorded during a 150-second application of solvent. Changes in membrane voltage were recorded, whereas the cell was perfused for 600 seconds with either solvent (0.02% DMSO in DRG neurons and 0.08% DMSO in SDH neurons), APAP (100 μM), AM404 (10 μM), or NAPQI (1 μM). To evoke action potentials, current injections in 5 equal increments were made for a period of 2 seconds each, once every 10 seconds. Membrane excitability was evaluated twice, once before the evaluation of the membrane voltage (in presence of solvent) and once subsequent to that (in presence of DMSO, APAP, AM404, or NAPQI). (A) Shows representative traces in the presence of solvent (ctrl, black trace), and NAPQI (gray trace) after 600 seconds in DRG (left panel) and SDH (right panel) neurons. (B) Displays the relation between amplitudes of injected currents and the resulting number of action potentials (no of spikes) under control conditions in 24 DRG (left) as well as 24 SDH (right) neurons. (C) Presents time courses of changes in membrane voltage (Δ voltage) and corresponding changes in action potential numbers (no. of spikes) for DRG (left panels) and SDH (right panels) neurons. The gray-shaded areas indicate the presence of the respective compound: DMSO (DM), paracetamol (APAP, AP), AM404 (AM), and NAPQI (NA). Before the application of a compound, the baseline was recorded in solvent (0.02% DMSO for DRG and 0.08% DMSO for SDH neurons). (D) Exhibits statistical analyses of changes in membrane voltage (Δ voltage) and number of action potentials (normalized spikes) in presence of the respective compounds (DMSO, DM; APAP, AP; AM404, AM; and NAPQI, NA). *, **Significant differences vs solvent after 600 seconds of perfusion at *p* < 0.05 and *p* < 0.01, respectively (Kruskal–Wallis test, Dunn's multiple comparison post hoc test, n = 6). NAPQI, N-acetyl-p-benzoquinone imine.

Small-diameter DRG neurons were classified by their current response to capsaicin (0.3 μM, applied for 15 seconds) recorded at −70 mV. Only capsaicin-positive DRG neurons were considered for further investigation, whereas all types of neurons from SDH were included in the analysis. To test for membrane excitability, 5 depolarizing currents (each lasting for 2 seconds with 8-second intervals; duration of the entire stimulus protocol 50 seconds) with increasing amplitudes were injected. Because membrane excitability varies considerably between single DRG and SDH neurons,^42,54^ initial amplitudes and the subsequent equal increments were designed individually for each neuron to achieve evenly increasing spike numbers with incremental current amplitudes. The initial amplitude was chosen to elicit at least one action potential; the number of action potentials elicited by the highest current amplitude was not permitted to be lower than that induced by any preceding less intense current injection. The resulting current spike relation is shown for both types of neurons in Figure 1B. The chosen current injection protocol was maintained for all recordings made within one cell. Baseline values for membrane excitability were recorded in presence of solvent. Thereafter, compound-mediated changes in membrane voltage were determined within the same cell, first by recording baseline values in presence of solvent for 150 seconds, followed by drug application (paracetamol, AM404, or NAPQI) for 600 seconds. Then, membrane excitability was tested again in presence of the respective compound. In current clamp recordings where linopirdine (30 µM) was applied, the resting membrane voltage of each cell was individually readjusted to its original value by current injection 120 seconds after the start of linopirdine application and 140 seconds before the start of NAPQI exposure.

Currents through K_V_7 channels were evoked at a holding voltage of −30 mV. Once every 30 seconds, the cells were hyperpolarized to −70 mV for a period of 1 second, which causes a slow closing of K_V_7 channels; differences in amplitudes 20 ms after the onset and 20 ms before the end of these voltage steps were taken as a measure of such currents. Baseline values were recorded for a period of 120 seconds in presence of solvent followed by a 10-minute perfusion with the different compounds and varying concentrations of NAPQI. In the latter case, this was followed by a washout with solvent for a period of 180 seconds. Finally, XE991 (3 μM) was perfused for a period of 180 seconds in all current recordings to verify the recording of currents through K_V_7 channels.

### 2.6. Electrophysiological experiments in spinal cord slices

A single slice was transferred to the recording chamber, where it was continuously superfused at a rate of 3 to 4 mL·min^−1^ with oxygenated recording solution. The incubation solution contained (in mM) the following: NaCl (95), sucrose (50), NaHCO_3_ (26), glucose (15), MgSO_4_ (7), KCl (1.9), KH_2_PO_4_ (1.2), CaCl_2_ (0.5), adjusted to pH 7.4, measured osmolarity 310 to 320 mOsmol·L^−1^. The recording solution was identical to the incubation solution except for (in mM) NaCl (127), CaCl_2_ (2.4), MgSO_4_ (1.3), and no sucrose. All recordings were conducted at room temperature (20-22°C). Superficial dorsal horn neurons were visualized with Dodt infrared optics^10^ using a 40×, 0.80 NA water immersion objective on an Olympus BX50WI upright microscope (Olympus, Japan). Lamina I was identified as the area located within a distance of less than 20 μm to the white matter. Only lamina I neurons were considered for experiments and recorded in the whole-cell patch clamp configuration with glass pipettes (2-4 MΩ) filled with internal solution (in mM): potassium gluconate (120), KCl (20), MgCl_2_ (2), HEPES (20), Na-GTP (0.5), Na_4_-EGTA (0.5), Na_2_-ATP (2), and phosphocreatine disodium salt hydrate (7.5); pH was adjusted to 7.28 with KOH, measured osmolarity 295 to 310 mOsmol·L^−1^. The patch pipettes were pulled on a horizontal micropipette puller (P-87; Sutter Instruments, Novato, CA) from borosilicate glass (Hilgenberg GmbH, Malsfeld, Germany). Voltage-clamp recordings were made at a holding potential of −70 mV using an Axopatch 700B patch-clamp amplifier and the pCLAMP 10 acquisition software package (both Molecular Devices, Union City, CA). Signals were low-pass filtered at 2 to 10 kHz, sampled at 20 kHz. The resting membrane potential was measured immediately after establishing the whole-cell configuration. Only neurons with a resting membrane potential more negative than −50 mV were used for further analysis. Membrane resistance, membrane capacitance, and series resistance were calculated from the averaged reaction to 20 consecutive hyperpolarizing voltage steps from −70 to −80 mV for 100 ms. Neurons with a calculated series resistance of more than 30 MΩ were excluded from further analysis.

Excitatory postsynaptic currents (EPSCs) were evoked by stimulating the dorsal root afferents using a suction electrode with an isolated current stimulator (A360; World Precision Instruments, Sarasota, FL). After determining the threshold value to elicit an EPSC, 2 consecutive pulses (0.1-ms pulse width, 300 ms delay) were given at 15-second intervals. The stimulation intensity was set at 200% of the threshold value and ranged from 2.0 to 8.2 mA (total mean of 4.5 mA). The afferent input was classified as being C-fiber-evoked based on a combination of response threshold and conduction velocity. Only EPSCs with a response threshold of above 1 mA and a conduction velocity of ≤1 m*s^−1^ met those criteria.^44^ C-fiber-evoked EPSCs (eEPSCs) were considered monosynaptic by the absence of failures during 10 consecutive pulses at 2 Hz and a jitter in response latencies as low as 10%. Only these neurons were taken for further analysis. Series resistance was monitored throughout the experiment by a square pulse of −10 mV for 10 ms applied at 15-second intervals. If the series resistance changed by more than 20% during the experiment, neurons were discarded. Pictures of the recorded neurons were taken directly after establishing whole-cell configuration and again at the end of recordings. If cells showed signs of swelling or other unnatural alterations, recording was excluded from further analysis. NAPQI (10 µM) and CNQX (10 µM) were bath applied to a closed system of 10-mL recording solution. After 150 seconds of control recording, NAPQI was bath applied for 600 seconds followed by bath application of CNQX.

### 2.7. Data analysis and statistics

Data from all types of electrophysiological recordings were analyzed offline using Clampfit 10.4 (Molecular Devices) and GraphPad Prism 5 (GraphPad Software, La Jolla, CA). For quantification of synaptic strength, the peak amplitude of the eEPSCwas measured. The mean amplitude of 12 eEPSCs after 600 seconds of NAPQI application and 1200 seconds after CNQX application were compared with the mean amplitude of 12 eEPSCs before NAPQI application (control). Spontaneous EPSCs (sEPSCs) were obtained from the traces of the eEPSC recordings from 12 sweeps before (control), 12 sweeps after 600 seconds of NAPQI application, and 12 sweeps after 1200 seconds of CNQX application. Only the last 10 seconds of each sweep were used to avoid any impact of the evoked C-fiber stimulation. To calculate frequency (events per second; 120-second time frame) and amplitudes of sEPSCs an event template was created from the average of at least 20 events from each cell. An automated event detection was performed based on each template without post hoc correction.

Unless specified otherwise, all values are arithmetic mean ± SEM. n values reflect single cells in all electrophysiological experiments. Significances of differences were evaluated using a Kruskal–Wallis analysis of variance followed by a Dunn's multiple comparison post hoc test; for pair-wise comparisons, the Wilcoxon signed-rank test was used. The critical value for statistical significance was set at *P* < 0.05.

### 2.8. Ethics approval statement

This study was performed in accordance with the guidelines of “Good Scientific Practice” of the Medical University of Vienna and with the Austrian animal protection law (http://www.ris.bka.gv.at/Dokumente/BgblAuth/BGBLA_2012_I_114/BGBLA_2012_I_114.pdf) as well as the Austrian animal experiment bylaws (http://www.ris.bka.gv.at/Dokumente/BgblAuth/BGBLA_2012_II_522/BGBLA_2012_II_522.pdf), which implement the European directive (http://eur-lex.europa.eu/LexUriServ/LexUriServ.do?uri=OJ:L:2010:276:0033:0079: en:PDF). The experiments were performed ex vivo, wild-type animals were sacrificed, and their spinal cords and ganglia were used thereafter. §2 of the Austrian animal protection law (http://www.ris.bka.gv.at/Dokumente/BgblAuth/BGBLA_2012_I_114/BGBLA_2012_I114.pdf) states that such experiments do not require an approval of the institutional ethics committee.

## 3. Results

### 3.1. NAPQI, but not paracetamol or AM404, decreases the excitability of dorsal root ganglion and spinal dorsal horn neurons

To analyze effects of paracetamol and its metabolites on the excitability of DRG and SDH neurons, current clamp experiments were performed, and currents of evenly increasing amplitudes were injected to elicit action potential firing (Figs. 1A and B). Using this procedure, changes in excitability of DRG neurons by inflammatory mediators and analgesics^46,53^ have been determined in previous experiments.

In the perforated patch configuration, the resting membrane potential of DRG neurons amounted to −62.9 ± 1.5 mV (n = 24). Injection of neuron-specific sequences of 5 depolarizing currents (see Methods) with increasing amplitudes led to the current spike relation in Figure 1B. In the presence of either solvent (DMSO) or 100 µM paracetamol, no obvious changes in membrane potential were observed during a 10-minute exposure period. Likewise, the numbers of action potentials triggered by current injection before and at the end of this 10-minute exposure were comparable (Fig. 1C), and there was no difference between solvent and paracetamol (Fig. 1C). AM404 (10 µM), by contrast, elicited a membrane depolarization by 5.9 ± 2.6 mV, and this effect remained stable during a 10-minute exposure (Fig. 1C). The number of stimulation-evoked action potentials at the end of the application of AM404 was smaller than the number before drug application (Fig. 1C). Nevertheless, these AM404-dependent changes were not significantly different from those observed in conjunction with solvent (Fig. 1D). NAPQI (1 µM) caused a transient depolarization followed by a hyperpolarization (−9.9 ± 2.6 mV) that increased continuously over time (Figs. 1A and C). The number of action potentials in response to current injection was greatly reduced after exposure to NAPQI for 10 minutes (Fig. 1C). Both, the NAPQI-induced alterations in membrane potential and action potential firing were significantly different from the corresponding values obtained with solvent (Fig. 1D).

In SDH neurons, the average resting membrane potential was −72.4 ± 1.7 mV (n = 24), and the stimulation protocols used led to the current spike relation shown in Figure 1B. Drug effects to be observed were almost the same as in DRG neurons. Neither DMSO nor paracetamol (100 µM) affected any of the parameters determined (Fig. 1C), and there were no statistically significant differences between the results seen in these 2 conditions (Fig. 1D). AM404 (10 µM) led to a minor (2.5 ± 0.38 mV) depolarization that slowly developed during the 10-minute application period, but the number of current-induced action potentials at the end of drug exposure was the same as the number beforehand (Fig. 1C). All results obtained in AM404 were not significantly different from those in solvent (Fig. 1D). NAPQI (1 µM) caused a −8.1 ± 1.0 mV hyperpolarization in SDH neurons that evolved slowly (time to maximum approximately 7 minutes; Figs. 1A and C), and, in parallel, action potential firing in response to current injection was largely reduced (Figs. 1A and C). The values of both changes, membrane hyperpolarization and action potential reduction, were significantly different from those obtained with solvent (Fig. 1D). Taken together, these data show that NAPQI, but not paracetamol or AM404, causes membrane hyperpolarization and reduces excitability in both, DRG and SDH neurons.

### 3.2. NAPQI, but not paracetamol or AM404, enhances currents through K_V_7 channels in dorsal root ganglion and spinal dorsal horn neurons

**Figure 2.**
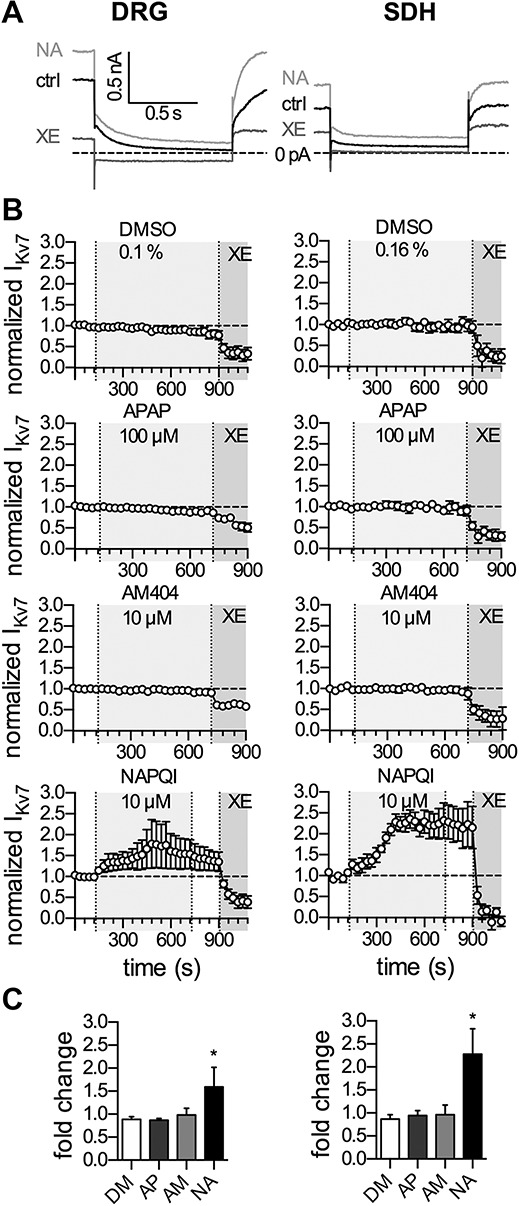
Effects of APAP, AM404, and NAPQI on currents through K_V_7 channels in rat DRG and SDH neurons. Currents through K_V_7 channels were recorded in perforated voltage-clamp mode. The cells were clamped to −30 mV and hyperpolarized to −70 mV for a period of 1 second, once every 30 seconds. The difference in amplitude determined at the onset and end of this hyperpolarization-induced current relaxation was taken as the readout of K_V_7 channel activity. Baseline current levels in presence of solvent (DM, 0.1% DMSO in DRG neurons, 0.16% DMSO in SDH neurons) were recorded for 120 seconds, followed by application of paracetamol (APAP, AP, 100 μM), AM404 (AM, 10 μM), or NAPQI (NA, 10 μM) for 600 seconds. (A) A 180-second washout period followed the application of NAPQI; XE991 (3 μM) was applied for 180 seconds at the end of all recordings. All amplitudes were normalized to the average of the first 5 current amplitudes. (A) Displays representative K_V_7 current traces in the presence of solvent (ctrl, black trace), NAPQI (NA, light gray trace), and XE991 (XE, dark gray trace) in DRG (left panel) and SDH (right panel) neurons. (B) Shows time courses of K_V_7 currents normalized to the first control values in the presence of the respective compounds in DRG (left panel) and SDH (right panel) neurons indicated by the light gray-shaded area. Solvent (DMSO 0.1% for DRG and DMSO 0.16% for SDH neurons) was present in absence of the respective compounds. XE991 (3 µM) was applied for 180 seconds at the end of all recordings, indicated by the dark gray area, to verify the recording of currents through K_V_7 channels. (C) Exhibits the statistical analysis of normalized K_V_7 currents (fold change) after 600 seconds of application of the indicated compounds (DMSO, DM; APAP, AP; AM404, AM; and NAPQI, NA) in DRG (left panel) and SDH neurons (right panel). *Significant differences vs solvent (DM) at *p* < 0.05 (Kruskal–Wallis test, Dunn's multiple comparison post hoc test) (n = 6). DRG, dorsal root ganglion; NAPQI, N-acetyl-p-benzoquinone imine; SDH, spinal dorsal horn.

One type of ion channel that regulates membrane potential, dampens neuronal excitability, and serves as target for analgesics is K_V_7.^3,11^ To reveal whether NAPQI influences membrane potential and excitability through an action on such voltage-gated K^+^ channels, neurons were clamped at −30 mV to activate K_V_7 channels. Channel deactivation was elicited by a 1-second hyperpolarization to −70 mV, and the amplitudes of the resulting current relaxation amounted to 147.0 ± 16.7 pA in DRG neurons (n = 24) and to 27.2 ± 5.7 pA in SDH neurons (n = 24; Fig. 2A). These current amplitudes were hardly affected by 10-minute application of solvent (DMSO), paracetamol (100 µM), or AM404 (10 µM; Fig. 2B). NAPQI (10 µM), by contrast, led to slowly arising increases in K_V_7 current amplitudes, and after 10-minute exposure, the amplitudes were increased more than 1.5-fold in DRG neurons and more than 2-fold in SDH neurons (Figs. 2A and B). In both cases, the NAPQI-induced augmentation of K_V_7 currents was irreversible after washout (Fig. 2B), and the relative changes in current amplitudes observed in NAPQI were significantly different from the corresponding values in solvent (Fig. 2C). Thus, NAPQI, but not paracetamol or AM404, causes irreversible increases in K_V_7 currents in both, DRG and SDH neurons.

### 3.3. Concentration dependence of the enhancement of K_V_7 currents by NAPQI

**Figure 3.**
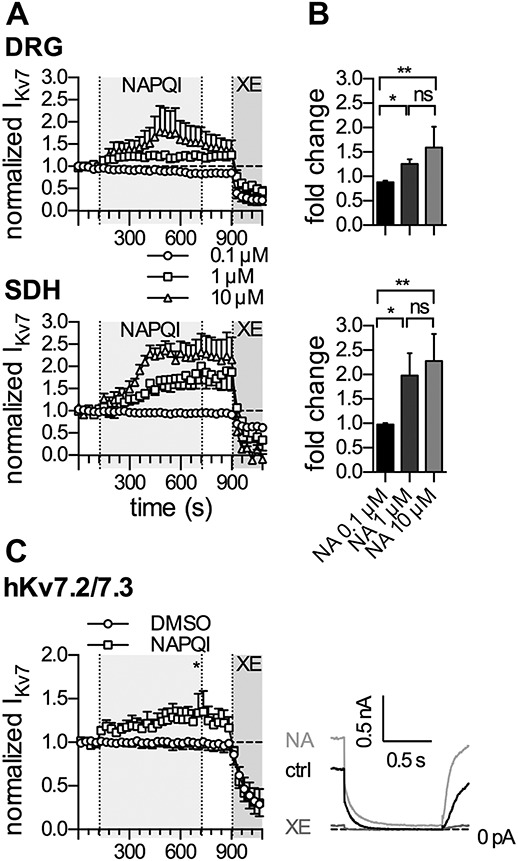
Effects of NAPQI on currents through native rat and recombinant human K_V_7 channels. Currents through K_V_7 channels were recorded in the perforated voltage-clamp mode. The cells were clamped to −30 mV and hyperpolarized to −70 mV for a period of 1 second, once every 30 seconds. The difference in amplitude determined at the onset, and end of this hyperpolarization-induced current relaxation was taken as the readout of K_V_7 channel activity. (A) Baseline was recorded in presence of solvent (0.1% DMSO in DRG neurons and tsA201 cells, or 0.16% DMSO in SDH neurons) for a period of 120 seconds, followed by different concentrations of NAPQI (0.1, 1, and 10 µM, as indicated) for 600 seconds, washout for 180 seconds and application of XE991 (3 μM) for 180 seconds. All amplitudes were normalized to the average of the first 5 current amplitudes in presence of the appropriate solvent. (A) Displays time courses of K_V_7 currents, normalized to control values, and changes in the presence of NAPQI applied at concentrations of 0.1 μM (circles), 1 μM (squares), and 10 μM (triangles) as indicated by the gray-shaded area in DRG (top panel) and SDH (bottom panel) neurons. (B) Exhibits the statistical analysis of K_V_7 currents, normalized to control values (fold change), at the end of a 600-second application of the indicated NAPQI concentration in DRG (top panel) and SDH (bottom panel) neurons. *, **Significant differences vs 10 µM NAPQI at *P* < 0.05 and *P* < 0.01, respectively. ns indicates no significant difference (Kruskal–Wallis test, Dunn's multiple comparison post hoc test) (n = 6). (C) Shows time courses of currents through heteromeric human K_V_7.2 and K_V_7.3 (hKv7.2/7.3) channels expressed in tsA201 cells (left panel). Cells were perfused with solvent (0.1% DMSO, circles; n = 6) or NAPQI (1 µM, squares; n = 6) for 600 seconds, as indicated by the gray-shaded area. The right panel shows representative current traces in presence of solvent (0.1% DMSO, ctrl, black trace), NAPQI (1 µM, NA, light gray trace), or XE991 (3 µM, XE, dark gray trace). *Significant difference of normalized K_V_7 current amplitudes: DMSO (0.91 ± 0.08) vs NAPQI 1 µM (1.29 ± 0.16) at *p* < 0.05 (Mann–Whitney test). DRG, dorsal root ganglion; NAPQI, N-acetyl-p-benzoquinone imine; SDH, spinal dorsal horn.

For irreversible drug actions, reliable concentration response curves are difficult to construct because all effects will be time- and concentration-dependent. To obtain information as to which NAPQI concentrations might be sufficient to elicit increases in K_V_7 currents, the results obtained with 10 µM NAPQI were compared with those of 10- to 100-fold lower concentrations. Neither in DRG, nor in SDH neurons, 0.1 µM NAPQI was able to induce current enhancement during a 10-minute period of drug application (Figs. 3A and B). Exposure to 1 µM NAPQI led to conspicuous current enhancement, but the rise in amplitudes reached its maximum in both types of neuron after 10 minutes only. In 10 µM NAPQI, for comparison, the maximal enhancement was achieved after 5 minutes in both cases (Fig. 3B). At the end of a 10-minute drug application, relative current amplitudes in 1- and 10 µM NAPQI were significantly different from those in 0.1 µM NAPQI, but not from each other (Fig. 3B). Hence, NAPQI concentrations as low as 1 µM are sufficient to irreversibly augment currents through K_V_7 channels in DRG and SDH neurons.

### 3.4. NAPQI enhances currents through recombinant human K_V_7.2/K_V_7.3 channels

To reveal whether K_V_7 channels in humans might be modulated by NAPQI in the same way as channels endogenously expressed in rat neurons, human K_V_7.2 and 7.3 subunits, the predominating combination in DRG neurons,^3,11^ were co-expressed in tsA201 cells. Current responses evoked by the pulse protocol described above were not affected by the solvent (DMSO) but were enhanced in an apparently irreversible manner by 1 µM NAPQI. The relative changes in current deactivation amplitudes at the end of a 10-minute exposure to NAPQI were significantly different from the corresponding values in solvent (Fig. 3C). Hence, recombinant human K_V_7.2/K_V_7.3 channels are about as sensitive towards NAPQI as K_V_7 channels expressed in rat DRG neurons.

### 3.5. The NAPQI-dependent decrease in excitability of dorsal root ganglion and spinal dorsal horn neurons relies on K_V_7 channels

**Figure 4.**
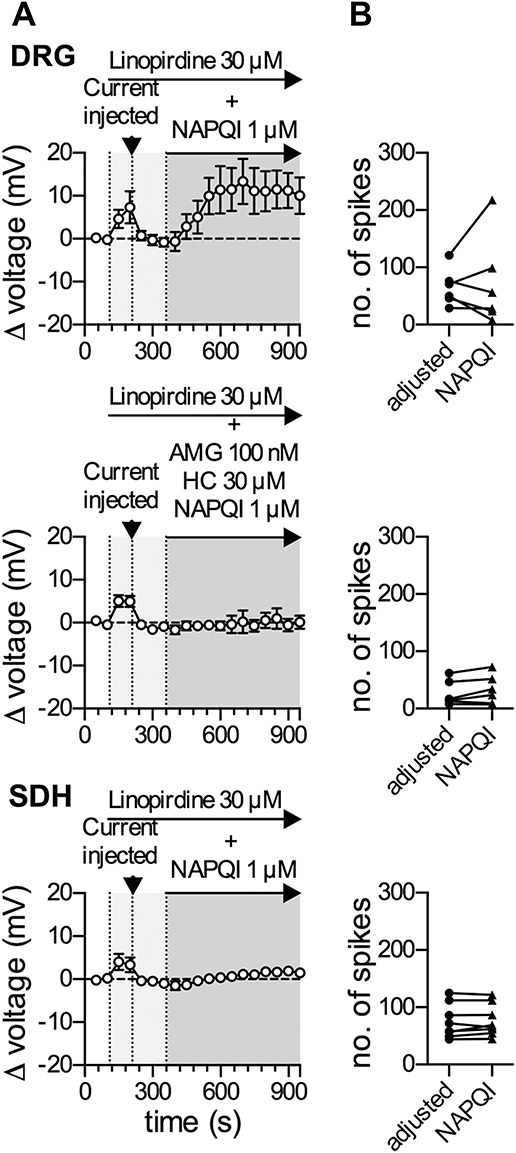
Linopirdine blocks the NAPQI-induced reduction in excitability and hyperpolarization in rat DRG and SDH neurons. Membrane voltage and excitability were recorded in perforated current-clamp mode. Baseline values for membrane potential and excitability were determined during a 100-second application of solvent (0.02% DMSO in DRG neurons and 0.08% DMSO in SDH neurons). This was followed by application of linopirdine alone (30 μM) for 150 seconds; the linopirdine-evoked depolarization was then compensated individually for each cell by current injection (indicated by the arrowhead in A) and a stable baseline was recorded for 150 seconds in the continuous presence of linopirdine. In DRG neurons, either NAPQI (1 µM) alone or in combination with the TRPV1 blocker AMG 9810 (100 nM) and the TRPA1 blocker HC 030031 (30 μΜ) was added for 600 seconds as indicated by the dark gray area. In SDH neurons, only NAPQI (1 µM) was present during the 600-second period indicated by the gray area. Membrane excitability was tested twice by injecting 5 equal increments of currents for a period of 2 seconds, once every 10 seconds, before and after the application of NAPQI for 600 seconds. (A) Shows time courses of changes in membrane voltage (left) in DRG (top) and SDH (bottom) neurons in the presence of linopirdine and with addition of NAPQI, either alone (top and bottom) or together with TRP blockers (middle; n = 6 for DRG and n = 8 for SDH neurons). (B) Shows the total number of action potentials in presence of linopirdine with compensated membrane potential before (adjusted, black circles) and at the end of a 600-second application of NAPQI (black triangles) either alone (top and bottom) or together with TRP blockers (middle; n = 6 for DRG and n = 8 for SDH neurons; *p* > 0.05 in each case, Wilcoxon matched-pairs signed rank test). DRG, dorsal root ganglion; NAPQI, N-acetyl-p-benzoquinone imine; SDH, spinal dorsal horn.

To reveal whether changes in membrane potential and excitability, on the one hand, and K_V_7 current enhancement, on the other, were causally related to each other, additional current clamp experiments were performed. Administration of the K_V_7 channel blocker linopirdine (30 µM) led to a slight (up to 8 mV) depolarization in both, DRG and SDH neurons (Fig. 4A). Subsequently, the membrane voltage was repolarized back to initial values through current injection in the continuing presence of linopirdine. Under these conditions (original membrane voltage, but K_V_7 channels blocked), 1 µM NAPQI failed to induce membrane hyperpolarization in both types of neuron: in SDH neurons, NAPQI did not induce any major alteration in membrane voltage (+1.5 ± 0.8 mV; Fig. 4A bottom trace); and in DRG neurons, by contrast, NAPQI led to a depolarization by ≥10 mV (Fig. 4A top trace). In both types of neuron, stimulation-evoked firing of action potentials remained unaltered despite a 10-minute exposure towards 1 µM NAPQI (Fig. 4B). Thus, when K_V_7 channels are blocked, NAPQI fails to cause membrane hyperpolarization or to reduce neuronal excitability.

In DRG neurons, NAPQI is known to activate TRPA1^1^ as well as TRPV1^13^ channels, and opening of these cation channels can be expected to cause depolarization. To reveal whether the NAPQI-induced depolarization in DRG neurons with blocked K_V_7 channels was mediated by these 2 types of TRP channels, the APAP metabolite was applied again together with the TRPV1 blocker AMG 9810 (100 nM)^16^ and the TRPA1 blocker HC 030031 (30 μΜ).^35^ This concentration of AMG 9810 reduced currents evoked by 0.3 µM capsaicin (TRPV1 agonist) in DRG neurons from −1.01 ± 0.58 nA down to −0.01 ± 0.01 nA (n = 3), whereas 30 µM HC 030031 diminished currents induced by 5 µM allyl isothiocyanate (TRPA1 agonist) from −0.71 ± 0.24 nA down to −0.05 ± 0.01 nA (n = 3). With the 2 TRP channels plus K_V_7 channels being blocked, NAPQI failed to cause alterations in the membrane voltage of DRG neurons (Fig. 4A; middle trace), and action potential firing remained unaffected again (Fig. 4B). Hence, when K_V_7 channels of DRG neurons are blocked, NAPQI may cause membrane depolarization through TRPV1 and TRPA1 channels but does not affect action potential firing.

### 3.6. NAPQI does not affect synaptic transmission between dorsal root ganglion and spinal dorsal horn neurons

**Figure 5.**
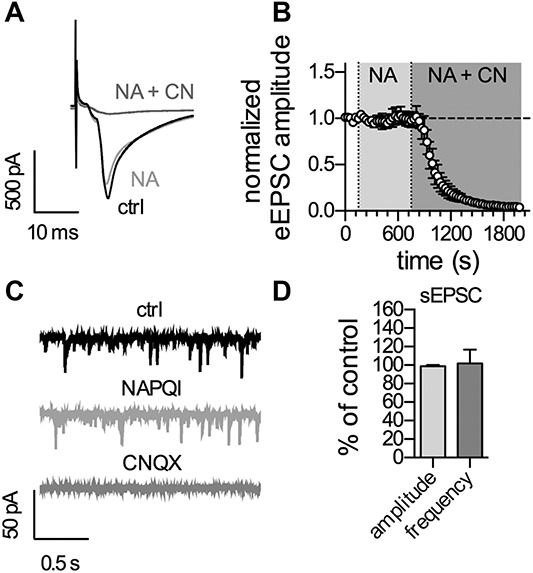
Application of NAPQI has no effect on synaptic activity in the rat spinal dorsal horn. Recordings were performed in lamina I neurons with monosynaptic C-fiber input in transversal spinal cord slices. (A) Shows original traces of C-fiber-eEPSCs recorded before and at the end of application of either NAPQI (NA) or NAPQI plus CNQX (NA + CN). (B) Displays the time course of normalized C-fiber-eEPSC amplitudes in lamina I neurons. Baseline was recorded for 150 seconds followed by application of NAPQI (10 µM) for 600 seconds (n = 7, *p* > 0.05 at the end of 600 seconds NAPQI compared with baseline). Bath application of CNQX (10 µM) blocked synaptic transmission at C-fiber synapses (n = 7, *P* < 0.05, one-way ANOVA at the end of 1200-second NAPQI + CNQX vs baseline). (C) Shows representative traces of sEPSCs at a holding potential of −70 mV before (top; ctrl), at the end of NAPQI application (middle), and at the end of NAPQI + CNQX (bottom). (D) Depicts the statistical analysis of the effects of NAPQI on amplitudes and frequencies of spontaneous EPSCs (sEPSCs) in the same lamina I neurons as in (B) determined at the end of 600-second NAPQI application (n = 7; *p* > 0.05 in both, one-sample *t* test). ANOVA, analysis of variance; NAPQI, N-acetyl-p-benzoquinone imine; sEPSCs, spontaneous excitatory postsynaptic currents.

Drug-induced changes in the gating of neuronal K_V_7 channels may lead to alterations in presynaptic transmitter release.^33,41^ Therefore, synaptic transmission between DRG and SDH neurons was investigated using transversal spinal cord slices with adherent dorsal roots. Monosynaptic C-fiber–eEPSCs were recorded from lamina I neurons as described previously.^28^ Bath application of 10 µM NAPQI failed to affect amplitudes of such eEPSCs, whereas the subsequent bath application of 10 µM CNQX completely blocked synaptic transmission between primary afferent C fibers and lamina I neurons (Figs. 5A and B). In addition, frequencies and amplitudes of spontaneous EPSCs remained unaltered in the presence of NAPQI (Figs. 5C and D). This reveals that the NAPQI-induced changes in neuronal excitability in DRG and SDH neurons are not accompanied by alterations in synaptic transmission.

### 3.7. NAPQI abrogates bradykinin-induced K_V_7 channel inhibition

**Figure 6.**
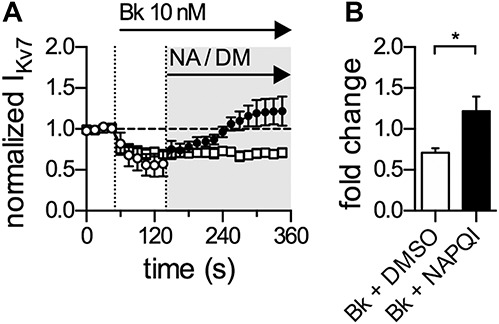
NAPQI abolishes bradykinin-mediated inhibition of currents through K_V_7 channels in rat DRG neurons. Currents through K_V_7 channels were recorded in the perforated voltage-clamp mode. The cells were clamped to −30 mV and hyperpolarized to −70 mV for a period of 1 second, once every 15 seconds. The difference in amplitude determined at the onset and end of this hyperpolarization-induced current relaxation was taken as the readout of K_V_7 channel activity. (A) Shows a time course of normalized currents through K_V_7 channels recorded in DRG neurons. Baseline current levels in presence of solvent (0.01% DMSO; DM) were recorded for 45 seconds followed by application of bradykinin (Bk, 10 nM) in the continued presence of solvent (empty circles and squares). Ninety seconds after the onset of Bk application, solvent was either replaced by NAPQI (NA, 1 µM, filled circles), or continuously coapplied together with bradykinin as indicated by the gray-shaded area. All amplitudes were normalized to the average of the first 4 current amplitudes in presence of solvent only. (B) Presents the statistical analysis of normalized K_V_7 currents (fold change) after 210 seconds of co-application of either Bk + solvent or Bk + NAPQI. *Significant differences vs solvent at *p* < 0.05 (Mann–Whitney test) (n = 6). DRG, dorsal root ganglion; NAPQI, N-acetyl-p-benzoquinone imine.

Bradykinin is a prototypic inflammatory mediator triggering hyperalgesia and allodynia through actions on an appropriate metabotropic receptor expressed by DRG neurons. The underlying mechanisms involve a G protein–dependent inhibition of K_V_7 channels.^4^ We therefore tested whether NAPQI might counteract this proalgetic mechanism. Application of 10 nM bradykinin (Bk) decreased K_V_7 current amplitudes by about 40% (Fig. 6A), and this inhibition remained stable during prolonged (up to 5 minutes) application of the peptide in the presence of solvent (Fig. 6A). However, when NAPQI (1 µM) was present, K_V_7 current amplitudes returned to control values within 2 minutes and thereafter even exceeded the amplitudes determined before the application of bradykinin (Fig. 6A). Accordingly, K_V_7 currents determined in the presence of bradykinin plus NAPQI were significantly larger than those in the presence of bradykinin plus solvent (Fig. 6B). Hence, NAPQI counteracts the G protein–dependent inhibition of K_V_7 channels in DRG neurons by inflammatory mediators.

## 4. Discussion

The present results demonstrate that the paracetamol metabolite NAPQI reduces excitability and causes membrane hyperpolarization in DRG and SDH neurons, first- and second-order nerve cells in the pain pathway. This is the first evidence for a hyperpolarizing action of NAPQI in primary neurons. Before, NAPQI has been reported to activate TRPA1^1^ and TRPV1^13^ channels of DRG neurons, respectively. As neuronal TRP channels are permeable to, but rather nonselective for different cations, their activation results in depolarization rather than hyperpolarization.^50^ This mechanism explains why NAPQI led to a depolarizing response in DRG neurons, once K_V_7 channels were blocked by linopirdine (Fig. 4). In fact, depolarization of DRG neurons through gating of TRPA1 and/or TRPV1 channels may also impede action potential propagation.^1,47^ However, in the present experiments, NAPQI failed to significantly affect action potential firing in DRG neurons with K_V_7 channels being blocked (Fig. 4). This indicates that the NAPQI-dependent hyperpolarization was a mechanism decisive for the decrease in DRG neuron excitability. This correlation becomes even more obvious in SDH neurons: there, NAPQI hardly affected the membrane potential in the presence of a K_V_7 channel blocker and left membrane excitability unaltered. This lack of effect can be correlated with the restricted expression pattern described for TRPV1 channels in neurons of the spinal dorsal horn.^52,55^

The above findings obtained with linopirdine point towards K_V_7 channels as targets for NAPQI. In voltage-clamp recordings of K_V_7 channel deactivation currents in DRG and SDH neurons, NAPQI led to slowly developing and irreversible increases in current amplitudes, which were blocked by XE991, a selective K_V_7 channel blocker.^3^ Dorsal root ganglion neurons are well known to express K_V_7.2, K_V_7.3, and K_V_7.5 proteins and to display currents mediated by the respective channels.^3,11^ Akin effects were also observed with recombinant human K_V_7.2/7.3 channels. Together, these results confirm that NAPQI acts directly on members of this K^+^ channel family and adds another molecule to the list of well-established analgesics that target K_V_7 channels, which includes, for instance, flupirtine, diclofenac, meclofenamic acid, and celecoxib.^11,12,39^

The proalgetic actions of inflammatory mediators, such as bradykinin^31^ and nucleotides,^53^ involve a G protein–dependent inhibition of K_V_7 channels in DRG neurons. To learn whether NAPQI may enhance K_V_7 currents even when these were suppressed by inflammatory mediators, the paracetamol metabolite was applied in the continuing presence of bradykinin. The peptide reduced K_V_7 current amplitudes for prolonged periods, but the addition of NAPQI rapidly reverted this inhibition. Thereafter, current amplitudes in the presence of bradykinin plus NAPQI even exceeded control values determined before the application of the peptide. This shows that NAPQI can recover K_V_7 channel function even in the presence of inflammatory mediators as previously reported for other analgesic drugs that target these ion channels, such as flupirtine and retigabine.^30^

Functional characteristics of K_V_7 channels in the spinal cord are poorly investigated, although the expression of relevant proteins has been detected by immunohistochemistry.^9^ Intrathecal administration of modulators of K_V_7 channels has been found to alter spinal discharges in response to C-fiber stimulation, on the one hand, and to affect nocifensive responses, on the other hand.^6^ Moreover, in rat dorsal horn neurons, whether in dissociated primary cultures^26^ or in respective slices,^37^ currents with voltage dependencies typical of K_V_7 channels have been described, and these currents were either suppressed by activation of muscarinic receptors^37^ or enhanced by flupirtine,^26^ 2 pharmacological hallmarks of these channels. In the present experiments, the deactivation protocol for K_V_7 channels caused current relaxations in SDH neurons that were kinetically similar to those observed in DRG neurons but had much smaller amplitudes (27 vs 147 pA). Importantly, these current relaxations were completely abolished by the selective K_V_7 channel blocker XE991. Together with the results obtained with linopirdine (see above), another K_V_7 channel antagonist, this firmly establishes a functional role of K_V_7 channels in SDH neurons.

Having established that NAPQI affected both, first- and second-order nerve cells of the pain pathway, one comes across the question whether this paracetamol metabolite might alter synaptic transmission in between these neurons as well. As this cannot be investigated with dissociated neurons, experiments were performed on transversal spinal cord slices with adhering dorsal roots. However, neither monosynaptic C-fiber-evoked nor spontaneous EPSCs were altered by an application of 10 µM NAPQI for 10 minutes. The fact that even tenfold lower concentrations were sufficient to modulate membrane potential, excitability, and currents through K_V_7 channels in dissociated neurons indicates that this negative result cannot be explained by a mere lack of drug concentration being applied to slices as opposed to cultures. In the hippocampus, modulators of K_V_7 channels were found to regulate frequencies, but not amplitudes, of miniature EPSC and IPSCs, which demonstrates presynaptic regulation.^41^ In this brain region, K_V_7.2 and K_V_7.3 proteins were shown to be expressed on presynaptic nerve terminals.^8^ In the spinal cord, however, these channel proteins are rather concentrated at axon initial segments and nodes of Ranvier,^9^ but evidence for presynaptic K_V_7 channels is lacking.

Up to now, analgesic drugs such as fenamates and diclofenac were shown to enhance currents through neuronal K_V_7 channels in a reversible manner.^12,39^ By contrast, the effects of NAPQI on currents through K_V_7 channels were irreversible. This leads to the question of underlying mechanisms. NAPQI is known to readily react with cysteine residues of proteins.^7^ In line with such an action, NAPQI has been demonstrated to gate TRPV1 channels by targeting cysteine residues.^13^ Likewise, TRPA1 channels can be activated by NAPQI^1^ and other cysteine-modifying reagents, such as N-ethylmaleimide.^25^ Neuronal K_V_7 channels, in particular K_V_7.2, harbor several cysteine residues, and N-ethylmaleimide enhances currents through such channels through at least one of these cysteines. Moreover, the enhancement of K_V_7 channel currents by N-ethylmaleimide is irreversible^43^ as is the action of NAPQI shown above. Hence, by analogy, it seems reasonable to assume that NAPQI targets K_V_7 channels by conjugation with their cysteine residues. In this context, it should be noted that fenamates and diclofenac, in contrast to NAPQI, activate TRPA1 but block TRPV1 channels.^23^ This points towards differences in the underlying molecular mechanisms. In fact, a fenamate derivative has been shown to target voltage-sensing (rather than cysteine-rich) domains in K_V_7.2 as well as TRPV1 channels.^40^ It will be interesting to learn whether these apparent differences in the actions on K_V_7 and TRP channels between diclofenac, fenamates, and NAPQI can be translated into differences regarding their analgesic properties.

None of the actions of NAPQI were shared by paracetamol, the parental drug, or AM404, another active metabolite thereof. This raises the question as to whether NAPQI concentrations that are sufficient to act on K_V_7 channels become available during paracetamol therapy. Systemic administration of this analgesic leads to concentrations as high as 150 to 200 µM in plasma and up to 100 µM in cerebrospinal fluid.^36,51^ Five to fifteen percent of a paracetamol dose are metabolized towards NAPQI, which corresponds to calculated concentrations in the range of 10 µM. However, NAPQI has a very short half life because it readily reacts with sulfhydryl groups to give gluthathion and proteinyl cystein adducts.^34^ As a consequence, NAPQI concentrations cannot be measured reliably, but the relevant adducts have been detected in the central nervous system, particularly spinal cord,^1^ as well as the liver and other peripheral tissues.^38^ Moreover, the enzymes mediating the conversion of paracetamol to NAPQI, CYP2E1, and CYP3A4^29^ have both been shown to be expressed in the central nervous system,^2,15^ and the expression of CYP3A4 has been reported for human dorsal root ganglia as well.^18^ Taken together, these pieces of evidence suggest that K_V_7 channels in the pain pathway get exposed to NAPQI after paracetamol application, but it remains to be demonstrated that such a mechanism may contribute to paracetamol-induced analgesia.

In summary, this report unveils a previously overlooked direct inhibitory effect of the paracetamol metabolite NAPQI on neurons in the pain pathway, which relies on an activation of K_V_7 channels. Because paracetamol is converted to NAPQI not only in the liver, but also in other tissues including the CNS,^1,38^ this mechanism may be relevant for the recently discovered anticonvulsant properties^48,49^ and for the well-documented analgesic action of paracetamol.

## Conflict of interest statement

The authors have no conflict of interest to declare.
